# Florida Emergency Physicians 9^th^ Annual Symposium on Emergency Medicine, April 8-11 2009

**Published:** 2009

**Authors:** Dale S Birenbaum, Peter M DeBlieux

**Affiliations:** Academic Chairman, Program Director, Florida Hospital Emergency Medicine Residency Program, 7727 Lake Underhill Road, Orlando, FL 32822, USA; 1 LSUHSC Emergency Medicine Director of Faculty and Resident Development, New Orleans, LA, USA

**Keywords:** Quik clot, resuscitation, vasopressors, ventilator tidal volume

## Abstract

At the Florida Emergency Physicians Annual Symposium on Emergency Medicine, several important updates that could have a direct impact on patient care were presented by nationally recognized leaders in emergency medicine, critical care and trauma. All the topics presented were relevant to more than one specialty and included detailed discussions on optimal ventilator tidal volume, tissue oxygen saturation, ideal blood sugar levels, timing of intubation, appropriate blood transfusions and vasopressor use in the critical care patient. This article will focus on three aspects: Optimal ventilator tidal volume in the critical care patient, the use of a new hemostatic agent for hemorrhage control and vasopressor use in the early resuscitation of hemorrhagic shock.

## INTRODUCITON

At the Florida Emergency Physicians 9^th^ Annual Symposium on Emergency Medicine that took place at the Rosen Center between April 8 and 11, 2009 in Orlando, FL, several important updates that could have a direct impact on patient care were presented by nationally recognized leaders in emergency medicine, critical care and trauma.

All the topics presented were relevant to more than one specialty and included detailed discussions on optimal ventilator management, tissue oxygen saturation, ideal blood sugar levels, timing of intubation, appropriate blood transfusions and vasopressor use in the critical care patient.

The information was presented by Dr. Peter DeBlieux, Professor of Clinical Medicine, Director of Emergency Medicine Faculty and Resident Development, Louisiana State University Health Center; Dr. Andrea Gabrielli, Professor of Anesthesiology and Surgery and Medical Director, Cardiorespiratory Services, University of Florida College of Medicine; and Dr. Michael Rotundo, Chairman of Surgery at East Carolina's Pitt County Memorial Hospital.

The focus of the article will be on optimal ventilator management in the critical care patient, the use of a new hemostatic agent for hemorrhage control and vasopressor use in the early resuscitation of hemorrhagic shock.

## VENTILATOR MANAGEMENT

During the daily clinical practice of emergency medicine, we are constantly balancing new and incoming patients with unstable critical patients that require immediate attention. Understanding the basic pathophysiology of disease and incorporating new information into clinical decision making will aid patient care.

As an example, ventilator settings that are made in the emergency department can impact patient outcomes and are helping save the lives of our intensive care unit (ICU) patients. In their presentations on the management of the critically ill patient, Dr. Peter DeBlieux and Dr. Andrea Gabrielli point out the following information:

The lung's primary function is to add oxygen and remove carbon dioxide from the blood. In order for this to happen, the gas we breathe must be matched to the blood flowing through our lungs by the pulmonary capillary beds. Because the average minute alveolar ventilation (Va) for a healthy adult is 4 L/min and the resting cardiac output (Q) is 5 L/min, optimal ventilation/perfusion matching is 0.8 Va/Q. Optimal Va/Q matching is unlikely because the distribution of gas and blood varies across the lung fields for several reasons.[1]

In the past, during polio epidemics, “iron lungs,” which are negative pressure ventilators, were used to closely simulate normal breathing and allow the passage of air from the mouth to the alveoli with little effect on the cardiac output and with good Va/Q ratios. The use of iron lungs was not practical for routine care as they were difficult to apply, move and maintain and limited a physician's access to the patient's body.[1]

Currently, all modern ventilators utilize positive pressure mechanical ventilation and, unlike normal breathing, increase transpulmonary pressure, reduce venous return and, ultimately, lower cardiac output. Ventilated patients sometimes require peak airway pressures of 30 cm H_2_O or more. Normal systolic pulmonary arterial pressures seldom exceed 20-25 mmHg. Therefore, during positive pressure ventilation, if the intraluminal alveolar pressure (high volume) exceeds the pulmonary artery pressure, blood flow and gas exchange cannot occur until the alveolar pressure begins to fall below the pulmonary artery pressure during exhalation.[1]

Decreases in alveolar volume (low volume) can be caused by partial airway obstruction, surfactant depletion and alveolar edema, which can cause dysfunction and lead to hypoxemia and the Acute Respiratory Distress Syndrome or ARDS. During complete exhalation, alveolar volumes are also reduced and there is an increased work of breathing in trying to expand them to the ideal size and, therefore, the utilization of Positive End Expiratory Pressure (PEEP) helps prevent alveolar collapse. Using PEEP to keep more of the alveoli open is referred to as the open lung approach.[1]

Combining positive pressure mechanical ventilation with PEEP usually decreases shunting and improves oxygenation. In order to prevent ARDS and reduce morbidity and mortality, there is a tremendous amount of ongoing work and research on the best ventilator management approach to be taken for the management of critically ill patients and it appears that an approach looking at high- and low-volume injury is essential.

For many years, the concept of pulmonary barotrauma was limited to extra-alveolar air leaks. It is also clear that human lungs can be damaged internally by the ventilator with or without air leaks and this type of damage is known as ventilator-induced lung injury (VILI).

Traditional training in emergency medicine on mechanical ventilatory support calls for the following:

“Initially, the tidal volume is set at 10 ml/kg ideal body weight and the rate is adjusted accordingly. Sufficient time should be allowed for expiration. The peak airway pressure is maintained below 35-45 cm H_2_O to prevent barotrauma. The peak airway pressure appears to be related to barotrauma more than to the level of continuous positive airway pressure (CPAP). To reclaim lung volumes, PEEP or CPAP should be considered if the decreased pulmonary compliance prevents delivery of an adequate tidal volume or if hypoxemia persists despite 100% FIO_2_.” Tintinalli 6^th^ edition pg 118.

Dr. Peter DeBlieux points out the following: Raising PEEP progressively increases residual lung volumes and total lung volumes along with pressures. The combination of traditional tidal volumes 10 ml/kg and utilization of peak airway pressures instead of lower tidal volumes and the utilization of plateau pressure measurements place the patient at risk for VILI. These increasing pressures and relative volumes may inhibit venous return and injure the few remaining healthy lung units in the setting of diffuse pulmonary disease.

Increased lung volumes and plateau pressures then allow for the derecruitment of the healthy lung, which may become over distended creating new lung injury. He, therefore, recommends the following settings for patients placed on mechanical ventilation for respiratory failure in the presence of diffuse infiltrative lung diseases (ARDS, congestive heart failure (CHF) multilobar pneumonia, large pulmonary contusion):

1. Set the tidal volume at 6 cc/kg based on ideal body weight vs 12 cc/kg in these patients. It helps save their lives. This is based on conclusive data from the ARMA study that revealed the importance of low-volume ventilation in ARDS, with substantial improvement in several indices using 6 ml/kg rather than 12 ml/kg tidal volume.[2,3]
2. Serial increases in PEEP to achieve oxygenation goals should be accompanied by serial measurements of plateau pressures. Once plateau pressures are greater than 30 cm H_2_O, a consideration should be made for very small, 25-50 cc, reductions in tidal volumes to achieve plateau pressures less than 30cm H_2_O.

In order to aid in achieving good patient outcomes, Dr. DeBlieux and Dr. Gabrielli point out that it is not always necessary to maintain an oxygen saturation of 100% in the critical care patient. In an effort to achieve oxygen saturations of 100%, we are pushing patients too hard with increased airway pressures and tidal volumes in an effort to maintain oxygen saturations of 100%. Frequently, these patients still have adequate tissue oxygen saturation. Therefore, based on the oxygen hemoglobin dissociation curve, it is reasonable to consider oxygen saturation goals of 88-92% in those patients with an FIO_2_ of 100% in which there is no sign of cardiac or neurological evidence of ischemia. The use of tissue oxygen saturation devices is expanding rapidly throughout ICUs and has helped in confirming these findings.

Both Drs. Gabrielli and DeBlieux remind us to disconnect critically unstable patients from mechanical ventilation and to bag patients with 100% oxygen because this will give us the best chance to facilitate resuscitation and take control of the patient's ventilation. This simple maneuver helps to exclude the mechanical ventilator as the culprit creating the instability while the clinician explores the inciting event.

An additional point of information discussed was that the normal inspiratory to expiratory ratio is about 1:2 and inspiratory times longer than 1 s are poorly tolerated in awake patients. Longer inspiratory times can help reduce peak airway pressures but will require sedation. In patients who have significant airway obstruction, there may not be enough time for exhalation, which can result in auto-PEEP. The auto-PEEP can cause hemodynamic compromise. Therefore, pay attention to expiratory time in patients with all forms of clinical airway obstruction

## QUIK CLOT

Uncontrolled hemorrhage after both civilian and combat traumatic injury is the most common cause of potentially preventable death. These patients will often require transfusion to complement rapid surgical or angiographic hemostasis.[4] Many hemostatic products have been tested in an effort to control hemorrhage. There are currently two FDA-approved devices that are distributed in the battlefield, a chitosan-based dressing called Hemcon and Quik Clot.

The US Army currently distributes Hemcon and the US Navy and Marines distribute Quik Clot. Quik Clot is recommended by the US Navy to help control life-threatening external hemorrhage after routine means have failed. The prehospital military trauma life support course recommends pressure dressings to be applied to wounds and to escalate Hemcon or Quik Clot if this does not control the bleeding.[5]

Quik Clot is a manufactured granular mineral zeolite. It is an inert mineral product composed of oxides of silicon, aluminum, sodium and magnesium and small amounts of quartz. It acts as a molecular sieve and rapidly absorbs water in a nonchemical, physical reaction. The process generates heat but the primary mechanism of action effecting hemostasis appears to be the rapid absorption of water and concentration of platelets and clotting factors to the site of application that helps promote clot formation.[5]

Dr. Michael Rotundo reviewed a case series on the FDA-approved local hemostatic agent Quik Clot. The information was gathered from a study titled Quik clot Use in Trauma for Hemorrhage Control: Case series of 103 Documented uses by Peter Rhee *et al*.[5]

In the study, there were 103 documented cases of Quick Clot use: 69 by the US military in Iraq, 20 by civilian trauma surgeons and 14 by civilian first responders. There were 83 cases involving application to external wounds. There were 20 cases of intracorporeal, off label use by military and civilian surgeons.

The overall efficacy rate was 92%. All field applications by first responders were successful in controlling hemorrhage. The eight cases of ineffectiveness noted by physicians were in moribound patients with massive injuries and the application was felt to be used as a last resort. Aside for one case of burning at the site of the application, there was only one complication felt to be due to scar tissue formed on a ureter during intracorporeal use.

Dr. Rotondo expressed his support on the use of Quik Clot to help control bleeding in trauma patients as the guideline for its use in the military was well controlled, stating, “We are likely to see more of this in the future!”

## VASOPRESSOR USE IN EARLY HEMORRHAGIC RESUSCITATION

Damage control resuscitation strategies are being developed to help our trauma patients. Sometimes traditional approaches are challenged in order to improve care in the future. Research in controlling resuscitations is ongoing in order to help correct coagulopathies, acidosis and hypothermia that can occur in severely injured trauma patients.[6] During his presentation highlighting recent updates in the trauma literature, Dr. Michael Rotundo points out that damage control resuscitation advocates are transfusing patients earlier with increased amounts of plasma and platelets with the first units of packed red blood cells. This is occurring while simultaneously adjusting crystalloid use in the initial trauma resuscitation.

This has led to a renewed interest in the use of vasopressors for hemodynamic support during resuscitation. In a study from the University of Texas Southwestern Medical Center entitled “Early use of vasopressors after injury: Caution before constriction,” researchers looked at the effect of early vasopressor (EV) use and early aggressive crystalloid resuscitation (ECR) in severely injured trauma patients and the effect on mortality.[7]

Data on 921 significantly injured blunt trauma patients was used. Significantly injured patients had a mean injury severity score of 31 ± 13, with over 45% of the patients receiving greater than 6 units of blood within the first 24 h and 52% requiring surgery within 48 h, with an overall mortality rate of 12.3%.[7]

ECR therapy was defined in patients who had received greater than 16 L of crystalloid within the first 12 h and patients who had received greater than 20 L in the first 24 h post injury. EV use was defined in patients who had received the following vasopressors: Phenylephrine, norepinephrine, dopamine and vasopressin up to 12 and 24 h post injury. These vasopressors were studied because of their considerable vasoconstrictive effect verses others.

Patients who received EV therapy or aggressive ECR, in this analysis, were more severely injured with greater physiologic derangements, more frequently requiring interventions and intensive care. The crude mortality rate for no EV was 8.9% vs EV 34.5% and no aggressive ECR 11.2% vs aggressive ECR 17%.[7]

Despite having the overall crude mortality rates, the results were significant.

1. EV use within 12 h was associated with an 80% higher risk of mortality along with a 2x risk of mortality at 24 h.
2. ECR was also independently associated with a 40% reduction in mortality at 12 h in patients who were younger than 55 years.

Because this was a secondary data analysis, the authors point out that the study can only provide associations and does not prove causality. Therefore, patients who remain hypotensive despite aggressive resuscitation may require vasopressor support to maintain organ perfusion even early after injury and hemorrhagic shock.[7]

Dr. Rotundo stressed that these findings are limited to the early resuscitation phase and that the results should not be generalized. Further prospective randomized studies are required to determine when to use vasopressor support in early trauma resuscitation.[7]

## CONCLUSIONS

Based on the detailed discussions and information presented, the following clinical pearls can begin to be incorporated into the clinical arena:

1. Use ARDSnet tidal volumes of 6 cc/kg of ideal body weight for those patients suspected of acute lung injury and diffuse infiltrates presenting to the ED in respiratory failure.
2. Measure the plateau pressures initially and serially with increases in tidal volume or PEEP to maintain plateau airway pressures < 30 cm H_2_O.
3. In those patients who are hypoxic on 100% FiO_2_, consider oxygen saturation goals of 88-92% in those patients without cardiac or neurological evidence of ischemia.
4. During code situations and during mechanical ventilator troubleshooting, disconnect critically unstable patients from mechanical ventilation and bag patients with 100% oxygen.
5. Consider the use of Quik Clot to control bleeding that has not responded to direct pressure.
6. When vasoconstrictors are used in early trauma resuscitation, they should be used cautiously and only after aggressive crystalloid or colloid resuscitation has failed to maintain adequate tissue perfusion in severely injured patients with hemorrhagic shock.
